# Successful treatment of Hidradenitis Suppurativa with tofacitinib: two cases and a review of the literature

**DOI:** 10.1093/omcr/omad003

**Published:** 2023-03-25

**Authors:** Afsaneh Sadeghzadeh Bazargan, Arezoo Pashaei, Azadeh Goodarzi

**Affiliations:** Department of Dermatology, Rasool Akram Medical Complex Clinical Research Development Center (RCRDC), School of Medicine, Iran University of Medical Sciences, Tehran 6715847141, Iran; Department of Dermatology, Rasool Akram Medical Complex Clinical Research Development Center (RCRDC), School of Medicine, Iran University of Medical Sciences, Tehran 6715847141, Iran; Department of Dermatology, Rasool Akram Medical Complex Clinical Research Development Center (RCRDC), School of Medicine, Iran University of Medical Sciences, Tehran 6715847141, Iran

## Abstract

Hidradenitis Suppurativa (HS) is a major public health challenge affecting people globally, which is painful and the hard lumps under the skin are prone to infection. We aimed to investigate whether tofacitinib can help people with HS in a safe and effective way. In this study, we report two cases diagnosed with HS. Tofacitinib was used as a part of the treatment plan. The first patient received 5 mg of tofacitinib twice daily, 36 weeks, and the second one for 24 weeks. Clinical outcomes are described. The efficacy of tofacitinib in HS was confirmed in our study. The clinical characteristics of the patients improved after receiving tofacitinib. Lesions discharge significantly reduced, particularly in the axillary area. Tofacitinib may be useful as an adjuvant therapy when used in combination with other treatments. Further research in this area is required to improve our understanding of treatment with tofacitinib at HS.

## INTRODUCTION

Hidradenitis Suppurativa (HS), also known as Acne Inversa, is a chronic, painful and occlusive disorder that occurs on the skin [1]. The prevalence of HS is estimated to be approximately 1–4% worldwide [2]. Papules, pustules and nodules in the groin, axilla, under the breast and between the buttocks are all signs of an HS lesion [3]. HS is a complex disease attributed to genetic, environmental and lifestyle factors, an active immune system, smoking, bacterial infections and hormonal changes. Depression, anxiety, stress, social problems and low quality of life are frequently cited as psychological consequences of HS [4]. Androgens play a crucial role in the pathogenesis of HS. Apocrine sweat glands are found in the axilla, breasts, face, scalp and perineum. The sweat glands are stimulated by androgens and cause hair follicles to get clogged, which can cause painful bumps under the skin [5]. Lymphocytes, neutrophils and cytokines are related to inflammation and dilation of the hair follicles, leading to the accumulation of secretions, inducing infection and thickening the scars [6]. Inflammation is often indicative of the pathological progression of HS. HS can be treated with oral antibiotics, hormonal therapies, immunosuppressants, etc [7]. Adalimumab is most commonly used to treat the signs and symptoms of some inflammatory diseases [7]. Patients who initially respond to adalimumab develop resistance despite continued treatment and relapse, or the drug loses its effect. Compared with other therapies, switching to a biological medicine called tofacitinib, which is taken orally, can be successful and safe [8]. Tofacitinib is a novel medication for the treatment of HS, which has the potential to help with inflammatory diseases, such as ulcerative colitis, rheumatoid arthritis, nail psoriasis, ankylosing spondylitis, sarcoidosis and COVID-19 [9,14]. Inflammatory stimuli activate intracellular signaling pathways, which cause the production of inflammatory mediators, including tumor necrosis factor (TNF)-α, interleukin (IL)-1ß, IL-17 and interferon (IFN)-γ. JAK Inhibitors are biological agents currently being used for the treatment of some diseases. Tofacitinib is an effective oral JAK inhibitor that suppresses the inflammatory process by blocking the JAK-STAT signaling pathway [9]. In this series, we described our experience with two cases of extensive HS treated with tofacitinib.

## CASE REPORTS

**Figure 1:**
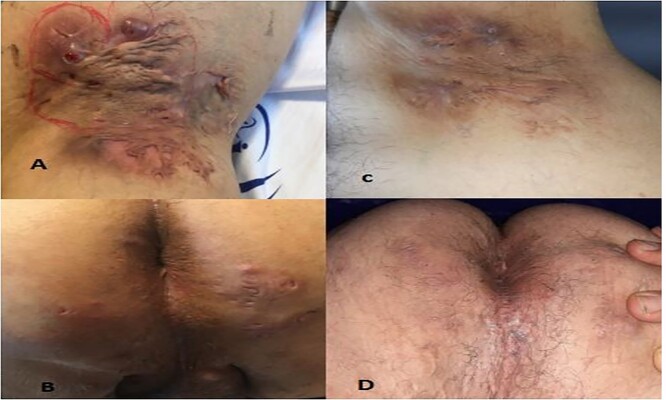
HS lesion in axillary and genital areas before the treatment (**A** and **B**); and after the treatment with tofacitinib (**C** and **D**).

Case 1: This is a case of a 40-year-old male smoker presented with a 15-year history of HS in the axillary and genital areas. The patient was admitted to Rassol Akram Medical Complex and received adalimumab and systemic antibiotics such as amoxicillin, although these treatments were unsuccessful. There was also no known family history of HS. In this case, a significant medical history revealed obesity with a BMI of 39.84. The patient was subjected to receiving 5 mg of tofacitinib twice a day. After 36 weeks of treatment, physical examination revealed that he was physically stable, inflammation had decreased, pain had subsided, and discharge and stiffness, especially in the axillary area, had decreased. Our results showed that tofacitinib was effective and successful in the treatment of HS (Fig. 1). During the therapy, the patient became infected with COVID-19 infection. Therefore, the drug was discontinued. The interruption of treatment resulted in a higher probability of disease recurrence, and the patient developed drug resistance to tofacitinib. Thus, he was treated with adalimumab after quarantine.

**Figure 2:**
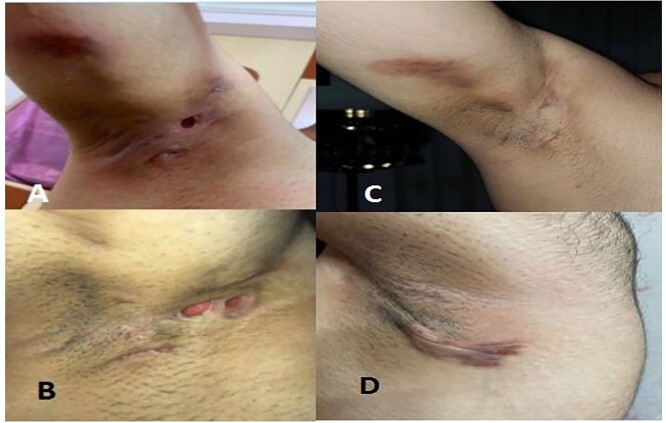
HS lesions in the axillary area before the treatment (**A** and **B**); and after the treatment with tofacitinib (**C** and **D**).

Case 2: This case was a 16-year-old boy with a history of HS, dating back to age 14, and characterised HS in axillary areas in advanced stage (Hurley stage 3) with pain and malodorous discharge symptoms. There was no record of disease and no positive family history. The patient had been treated with systemic antibiotics and Rifampin for 2 years, but the treatment was not effective enough and the disease was poorly controlled. The patient was referred to the hospital and prescribed tofacitinib (5 mg twice daily) in combination with Finasteride (1 mg daily). After 4 weeks of treatment, the main signs and symptoms such as pain, malodorous discharge and stiffness had decreased significantly by approximately 50%, which, in turn, improved his quality of life. At the end of the third month, the patient’s follow-up showed an improvement of about 90% in major signs and symptoms. However, liver function tests (LFT), including alanine transferase (ALT) and aspartate transferase (AST), showed a slight increase in the upper limit, and then liver ultrasound and gastrointestinal consultation were performed; we then continued treatment with tofacitinib 5 mg once a day. After we reduced the dose of tofacitinib, the LFT level did not seem to decrease. This is why the drug’s dose was reduced by half, with 5 mg taken every other day for 3 months, resulting in normal liver enzymes. After 6 months of treatment, the discharge, cavities and fistulas had disappeared, and axillary stiffness had decreased dramatically (Fig. 2).

## DISCUSSION

HS is an inflammatory disease that mainly affects women. Painful nodules and deep-seated inflammatory lesions in the skin occur in this disease [3]. Patients do not respond to the powerful first-line treatment, despite ongoing breakthroughs in HS treatment. Therefore, it is essential to make proper and safe use of the medicines for this disease [10]. Tofacitinib is a promising strategy to treat HS, and may provide new insights into the recovery and control of the disease [11]. The target of tofacitinib is to inhibit a protein called Janus kinase (JAK), which is involved in inflammation caused by the immune system and releases cytotoxic agents, TNF-α, IL-1β and IL-6 mediated by the JAK-STAT signaling pathway. This signaling system may be a therapeutic key if blocked [12]. Bacterial, mycobacterial, viral and other opportunistic infections and non-melanoma skin cancers including basal cell carcinoma, squamous cell carcinoma and Merkel cell carcinoma are the main potential adverse effects of JAK inhibitors, especially when used concomitantly with other immunosuppressants. Also, a decrease in the number of blood cells, hyperlipidemia and increased liver enzymes are the other side effects of these drugs [13].

**Table 1:** Summary of clinical characteristics of patients in our study and other studies

	This study		Cases reported in Kevin et al. [13]	
	Case 1	Case 2	Patient 1	Patient 2
Age	40	16	26	24
Gender	Male	Male	Male	Female
Hurley stage	III	III	III	III
Disease duration	15 years	2 years		
Anatomic region	Axillary areas, groins and penis	Axillary areas	Bilateral axillae, abdomen, bilateral inguinal folds	Inframammary, abdominal pannus, bilateral inguinal folds, perineum, buttocks, intergluteal cleft, posterior thighs
Prior therapies	Amoxicillin adalimumab	Systemic antibiotics, rifampin	Tofacitinib, cyclosporine and amoxicillin	Tofacitinib, mycophenolate mofetil (MMF), amoxicillin, triamcinolone ointment
Tofacitinib dose	5 mg 2 × daily	5 mg 2 × daily	5 mg 2 × daily	5 mg 2 × daily
Tofacitinib duration	9 months	6 months		
Tofacitinib Response	Improved	Improved	Improved	Improved

The clinical symptoms of HS improved in our patient after treatment with tofacitinib. In the first case, the patient became infected during treatment with COVID-19. The patient developed treatment resistance to tofacitinib after quarantine; therefore, he was given adalimumab to help him. The result revealed that the patient had a favorable response to tofacitinib. Similarly, 4 weeks after receiving tofacitinib, the second case showed a 50% improvement in the lesion, as well as a reduction in pain and discharge. Treatment with a lower dose of tofacitinib was prolonged for an additional three months due to an increase in LFT, with around 90% of effectiveness for this patient. The symptoms, medical history and clinical characteristics of two patients in this study are summarised in Table 1. Despite significant advances in the understanding, prevention, diagnosis and treatment of HS, it has remained a challenging issue. The use of tofacitinib as an alternative medicine represents a promising therapeutic approach in the treatment of HS. Unfortunately, there are insufficient data on the duration, action and maintenance of therapy with tofacitinib in the management of HS. The treatment of HS with tofacitinib in addition to other drugs may lead to a significant revolution in medicine. The results proved that tofacitinib is successful in the treatment of HS and improves patients’ quality of life, which is a promising development in the treatment of HS. Further clinical trials are required to prove that tofacitinib is a safe, effective and cost-effective treatment for the treatment of patients with HS. It can be concluded that JAK inhibitors provide a new perspective for the treatment of inflammatory diseases, especially HS.

Few trials have addressed the efficacy of tofacitinib as a first-line therapy in the treatment of HS. Tofacitinib, in combination with cyclosporine and amoxicillin, has been demonstrated to reduce injury of abdominal and axillary HS lesions, according to Kevin and colleagues (Table 1).

They showed that tofacitinib is an effective treatment for patients with HS, especially when they are resistant to other treatments [13].

This study investigated the effect of tofacitinib on HS lesions in the axillae and genital area after diagnosis HS. The treatment of HS with tofacitinib improves patients’ quality of life and contributes to HS control.

## Data Availability

Data resulted from this study are available from the corresponding author on reasonable request.
